# Dataset of Ash gourd plant leaf images for detection and classification

**DOI:** 10.1016/j.dib.2025.111997

**Published:** 2025-08-21

**Authors:** Nusrat Jahan, Md. Zahid Hasan

**Affiliations:** Health Informatics Research Lab, Department of Computer Science and Engineering, Daffodil International University, Daffodil Smart City (DSC), Birulia, Savar, Dhaka-1216, Bangladesh

**Keywords:** Ash Gourd Leaf Image, Machine learning, Plant disease recognition, Deep Learning, Agriculture

## Abstract

The Ash Gourd dataset is valuable since it was collected from the diverse regions within the district of Dhaka in Bangladesh. This dataset represents one of the first attempts to document, elicit, and categorize the health conditions of Ash Gourd (Benincasa hispida) plants in Bangladesh based on healthy samples, aphid plurality, downy mildew, leaf curl, and leaf miner-infested categories. Ash Gourd is one of the region's most important vegetables because of its nutritional and economic value; thus, it is essential to know diseases' manifestation in the improvement of agricultural productivity. The Ash Gourd dataset contains 2676 images, structured into the five categories of Healthy, Aphid, Downy Mildew, Leaf Curl, and Leaf Miner. All images in all categories are raw which can be used flexibly according to the needs of analysis and model training. Concretely, the Healthy class consists of 803 images, while the four other classes contain 1,873 images. This structured way of collecting data will, in turn, enable deeper analysis and help construct machine learning models for disease classification, hence providing worthy insights into Ash Gourd plant health.

Specifications Table

**Table:** 

Subject	Computer Science
Specific subject area	Computer Vision, Object Detection, Image Classification, Image Dataset, Machine Learning
Type of data	Images(.jpg) Raw
Data collection	• Healthy and infected images of Ash gourd leaves were collected separately. The infected images are further subcategorized into diseases, namely Aphid, downy mildew, Leaf Curl, and Leaf Miner. • Images were acquired using an 8MP, f/2.0 (telephoto), 1.0µm, PDAF, OIS, 3.7x optical zoom, and 5x hybrid zoom smartphone camera. • Those leaves were collected from 1 local Ash Gourd wooden mesh in October 2024.
Data source location	1. Sadullapur- Komolapur, Road Birulia Bridge, Dhaka 1216, Bangladesh. 2. Sinduria, Nayarhat, Savar, Dhaka, Bangladesh
Data accessibility	Repository name: Mendeley Data Data identification number: 10.17632/zj4th6xvdp.2 Direct URL to data:https://data.mendeley.com/datasets/zj4th6xvdp/2

## Value of the Data

• This dataset, introduced in Bangladesh for the first time, it provides insight into how the diseases of ash gourd leaves look under Bangladesh's climate, soil, and environmental conditions. The data also supports the development of disease recognition models that are highly effective for Bangladeshi farmers by enabling them to apply more precise interventions to specific disease patterns.
• The quality of data across the diverse diseases of leaves allows for the right and timely identification of powdery mildew and bacterial spot infections. Data-driven models enable early and accurate diagnosis, hence targeted treatments by farmers that minimize crop losses and reduce the use of broad-spectrum pesticides, which are costly and damaging to the environment.
• Automation of disease recognition through data-powered models reduces manual inspection time and labor costs. This gives farmers rapid diagnosis of their crops, hence providing timely interventions that stop diseases from spreading. The profits increase from the cost savings on labor and reduced production costs due to reduced applications of unnecessary pesticides.
• The dataset collected for disease recognition can serve as raw materials for further research in other areas like enhancements in computer vision, plant pathology, or AI agricultural tools. These could be the starting points where researchers build on refining diagnosis models, studying disease spread, and developing appropriate, more accurate, and disseminated tools in agriculture generally.

## 1 Background

Ash Gourd (Benincasa hispida) is considered a variety of winter melon and is grown as a nutritious vegetable in many Asian countries, including Bangladesh because it provides excellent nutrients and is tolerant of different climatic conditions. Simultaneously, being such an important part of national nutrition and local agriculture, Ash Gourd's studies about plant health and disease classification have not been significant in Bangladesh. Diseases of Ash Gourd include the infestation caused by aphids, downy mildew, leaf curl, and leaf miners, which have reduced yields in crops characterized by low-quality standards, hence making the farmers incur economic losses.

Due to a lack of documentation or analyses of disease patterns specific to Ash Gourd in Bangladesh, the research work has been challenging for researchers, agronomists, and practitioners. Thus, with this said, the dataset that we will be working on is unique, to the best of our knowledge, it is one of the pioneering efforts in Bangladesh focusing on the systematic collection and availability of visual data regarding the health of the Ash Gourd plant. This pioneering dataset is one of its kind, and it is aimed at creating ways for future research and development in Ash Gourd disease detection, hence making valuable insights available to support the local farmers and agricultural productivity in Bangladesh.

Agriculture in Bangladesh primarily faces similar problems due to its geographical vicinity with India and, therefore, almost analogous climatic conditions. Research conducted in the neighboring country, therefore, contributes a great deal as far as the study of viral diseases affecting gourds in Bangladesh [[1], [2], [3], [4], [5], [6]] is concerned along with their management strategies. For instance, studies on the Tomato Leaf Curl New Delhi Virus (ToLCNDV) [[6], [7]], which affects Ash Gourd and other cucurbits, demonstrate both the disease’s spread and management practices relevant to Bangladeshi agricultural contexts. Additionally, molecular studies of pest management techniques [8], such as integrated approaches against Bemisia tabaci, [9] provide actionable insights for managing similar threats in Bangladesh. This cross-border similarity in agricultural issues underscores the importance of regional research collaboration and knowledge exchange.

## 2 Data Description

**Fig. 1:**
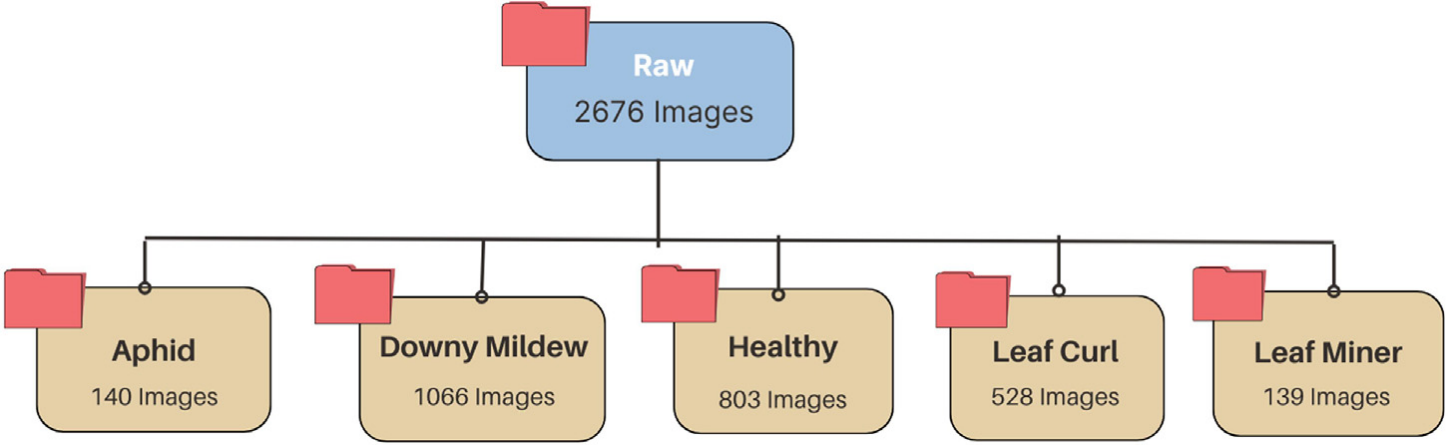
Folder hierarchy structure.

The Ash Gourd dataset is curated to assist in detecting and classifying diseases affecting Ash Gourd leaves. The classes of this dataset are primarily considered two categories: Healthy and Unhealthy. However, the class Unhealthy has been further divided into four specific types of diseases based on the type of infection or pest damage observed. Each category is in a separate folder with images showing typical visual symptoms associated with each kind of disease. The collection offers various ash gourd leaf images spanning five categories: Healthy, Aphid, Downy Mildew, Leaf Curl, and Leaf Miner [10]. This approach supports the structured classification of healthy leaves or those infected by specific diseases in the dataset; it is thus suitable for model training and validation tasks in plant disease detection. Table 1. depicts the distribution of images in each disease category and Fig. 1 shows folder hierarchy structure.

**Table 1:** Dataset sample distribution.

Name of the Diseases	Folder Name	No of images	Initial Image Resolution	Format
Aphid	Aphid	140	4512 × 6016px	.jpg
Downy mildew	Downy mildew	1066	4512 × 6016px	.jpg
Healthy	Healthy	803	4512 × 6016px	.jpg
Leaf curl	Leaf curl	528	4512 × 6016px	.jpg
Leaf miner	Leaf miner	139	4512 × 6016px	.jpg

### 2.1 Disease description

**Fig. 2:**
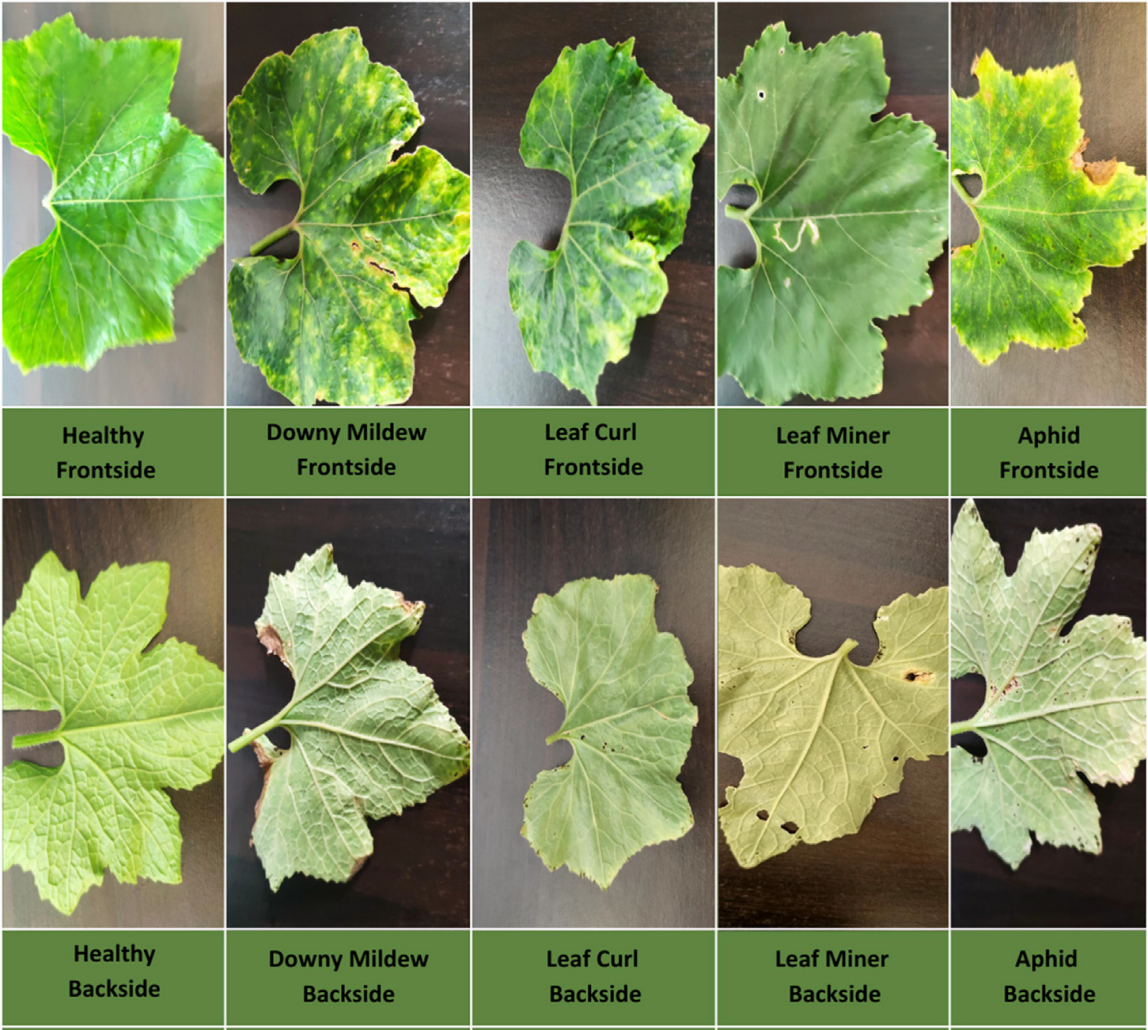
A sample of Ash Gourd leaf diseases and a healthy set.

Fig. 2 displays samples of Ash Gourd leaves affected by various diseases (Downy Mildew, Leaf Curl, Leaf Miner, Aphid) and a healthy set, showing both the front and back sides for each condition.

**Healthy:** This class consists of images of normal ash gourd leaves free of diseases, pests, and all other kinds of damage. The leaves are of normal shape and without any blemishes, which provides a basis for matching against the leaves of diseased ones. These include even coloration, no breakage of the leaf structure, no visible spots, discoloration, curling, or any kind of deformity. It is used as a control group to create contrast in classification, either for a healthy or diseased leaf.

**Downy Mildew:** This class includes images showing leaves infected with the fungal disease Downy Mildew. These infections thrive in moist and humid conditions and typically spread on the undersides of leaves, causing distinctive mottling and lesions. Yellow to pale green patches on the upper leaf surface and downy, greyish-white fungal growth on the underside. Affected areas may turn brown or necrotic as the disease progresses. Downy mildew reduces photosynthesis, weakens overall plant growth, and can severely affect crop yield if untreated [11].

**Leaf Curl:** Leaves in this category show leaf curl, often resulting from viral infections or pest vectors. The virus disrupts the leaf’s cellular structure, causing it to twist or curl. Puckering or twisting of leaf margins is often accompanied by discoloration and thickening of leaf tissue. Leaves may also appear leathery, distorted, or stunted. Leaf curl hinders photosynthesis and normal plant growth, potentially leading to stunted development and reduced fruit production [12].

**Leaf Miner:** This class represents leaves affected by leaf-mining insects. The larvae of these insects burrow between leaf layers, leaving visible feeding trails. Winding or blotchy marks across the leaf surface, often appearing as light-colored, translucent pathways. Affected areas may die off and turn brown as the damage advances. Leaf miner infestation reduces the photosynthetic area of the leaf, weakening the plant. Severe infestations can cause premature leaf drop and significantly lower plant productivity [13].

**Aphid:** These leaves are suffering from an infection caused by aphids. Aphids are tiny insects that feed on sap, causing serious damage to the leaf surface and generally affecting the plant's vitality. Small punctures, yellowing of leaves, and a mottled appearance. Leaves may appear distorted and develop sticky honeydew patches. Aphids can weaken the plant, reduce photosynthetic ability, and make the plant more vulnerable to secondary infections [12].

## 3 Experimental Design, Materials and Methods

Images in the dataset of ash gourd leaf have been captured using an 8 MP smartphone camera with a telephoto lens, f/2.0-aperture, 1.0µm pixel size, PDAF, and OIS. The camera supports up to 3.7 times optical zoom and 5 times hybrid zoom with enhanced digital processing and captures highly detailed pictures even when zoomed to further distances.

Images of leaves were captured at standardized and static distances of 20 to 90 cm to ensure close-up shots of the leaves while also giving slightly wider shots. Indeed, for it to be an image dataset that most accurately describes the real world, pictures are captured in a real-life environment under various natural lighting conditions, including sunlight, diffused light, and partial shade.

Since these are variations in imaging conditions, the model training and testing against disease symptoms and leaf positions will stand on robust ground, strengthening the standpoint of the dataset's applicability for trustworthy and adaptable ash gourd disease detection systems.

### 3.1 Data acquisition

The acquisition was made through a field collection of ash gourd leaf images using a high-resolution smartphone camera. Working with agricultural pathologists, we identified both the diseased and healthy ash gourd leaves, documenting them. On each visit to a session, pathologists helped diagnose the specific diseases affecting each leaf for proper identification and subsequent classification and labeling of images.

The images have been taken in natural daylight to represent the diseased and healthy leaves in their natural appearance. Photography of leaves at different angles and distances captures detailed features relevant to disease identification. Five distinct image categories were collected: Healthy and four disease-based classes: Aphid, Downy Mildew, Leaf Curl, and Leaf Miner.

**Fig. 3:**
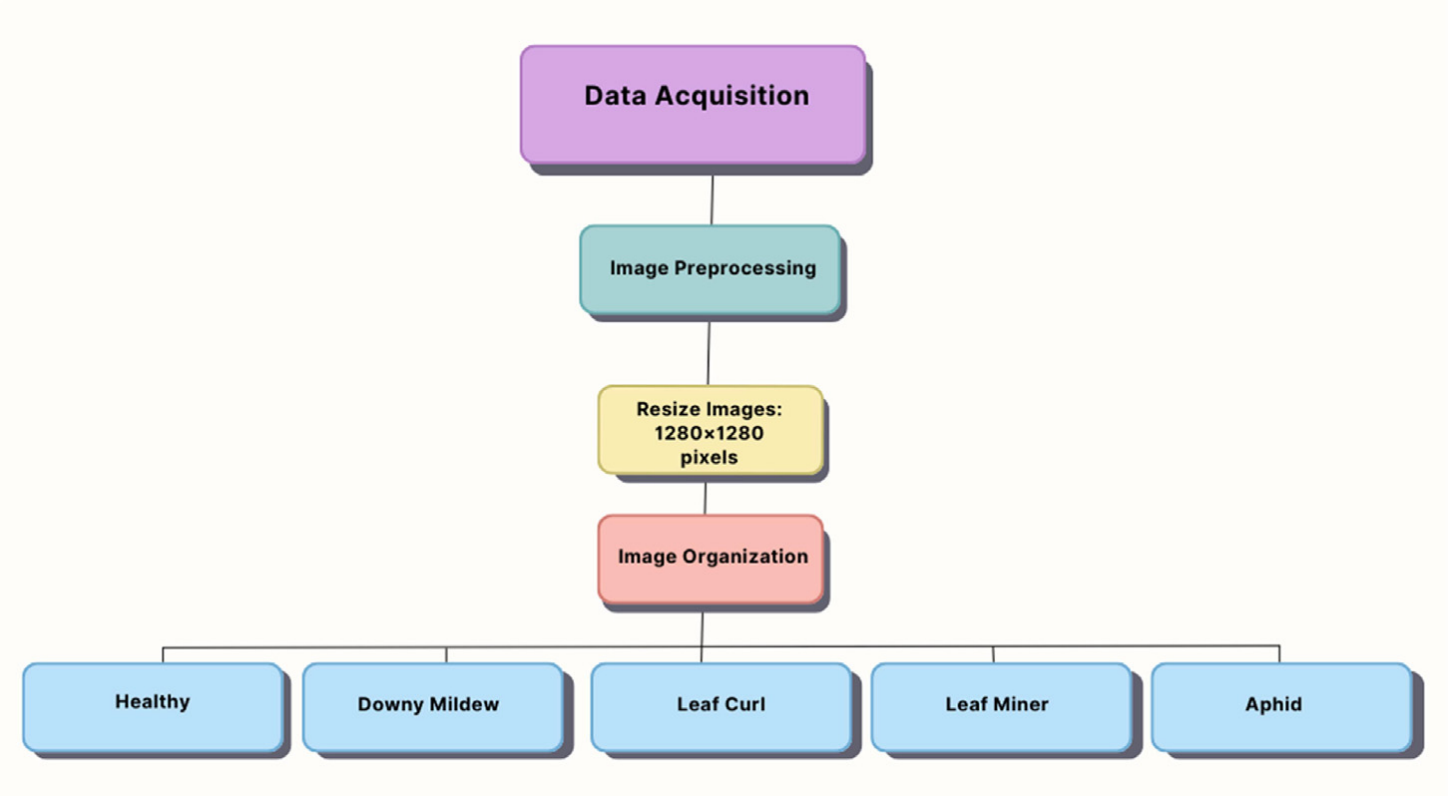
Data collection and processing pipeline for Ash gourd dataset.

Fig. 3 illustrates a data collection and processing pipeline for an Ash Gourd dataset, detailing stages of image acquisition, preprocessing, augmentation, and organization into disease categories.

### 3.2 Data pre-processing

It includes all the images in this dataset, resized to a fixed resolution of 1280 × 1280 pixels. This ensures that the images will all exactly have the same size, fitting into specific-sized requirements for the input of any neural network. The sizing of images should be uniform in deep learning models, which take input of specific dimensionality for their processing. Hence, it speeds up data processing and approves the model with better learning and generalizing of the pattern irrespective of the different sizes of images in the dataset.

## Limitations

• The dataset consists of raw, unprocessed images, allowing researchers the flexibility to apply their augmentation techniques tailored to their specific needs. This approach enables users to customize the dataset preparation process, ensuring compatibility with their chosen machine learning or deep learning models while minimizing unnecessary preprocessing overhead.
• This dataset comes from a relatively small area and might not represent the broader diversity of Ash Gourd plants from various climates and environmental conditions. Symptoms and leaf appearance can vary significantly due to regional factors like humidity, soil composition, and weather conditions. Consequently, this geographical limitation may mean that the dataset does not fully capture disease manifestations or health indicators relevant to other regions, affecting its applicability for studies or applications involving Ash Gourd plants elsewhere. The dataset's overall sample size is also quite limited, reducing its diversity and comprehensiveness regarding disease symptoms and plant health indicators within each category: healthy, aphid, downy mildew, leaf curl, and leaf miner.

## Ethics Statement

The authors have read and followed the ethical requirements for publication in Data in Brief and confirm that the current work does not involve human subjects, animal experiments, or any data collected from social media platforms.

## CRediT authorship contribution statement

**Nusrat Jahan:** Data curation, Conceptualization, Investigation, Methodology, Writing – original draft, Writing – review & editing. **Md. Zahid Hasan:** Supervision, Validation, Writing – review & editing.

## Data Availability

Mendeley DataAsh Gourd Leaf Healthy and Disease Dataset (Original data).
